# Benzodiazepine-resistant epilepsy: unraveling molecular mechanisms and developing multimodal therapeutic strategies

**DOI:** 10.3389/fneur.2025.1615079

**Published:** 2025-06-06

**Authors:** Yanqiu Huang, Yangfan Zhang, Yi Liang

**Affiliations:** ^1^School of Medicine, University of Electronic Science and Technology of China, Chengdu, China; ^2^Department of Neurology, Sichuan Provincial People’s Hospital, University of Electronic Science and Technology of China, Chengdu, China

**Keywords:** benzodiazepines, drug resistance, treatment strategies, epilepsy, seizure

## Abstract

Epilepsy is one of the most common nervous system diseases, which is characterized by recurrent seizures caused by abnormal neuronal discharges in the brain. Drug-resistant epilepsy (DRE) brings great challenges to clinical treatment. Benzodiazepines (BZDs), as the first-line treatment for acute seizures and Status Epilepticus (SE), are widely used because of their potent inhibitory neuromodulation by regulating *γ*-aminobutyric acid-A(GABA_A_) receptors. However, long-term use of BZDs may induce drug resistance, leading to a significant decrease in efficacy and increasing the difficulty of treatment. This study begins with the definition of BZDs-resistant epilepsy. It explores the underlying resistance mechanisms, including the down-regulation, decreased activity, and structural changes of GABA_A_ receptors, synapse and neural network remodeling, genetic variation in drug metabolism, and the effects of drug efflux mechanisms. In addition, combined with clinical practice and research progress, this study evaluates the effectiveness and potential of drug combination therapies, personalized treatments, and new treatment methods, highlighting the advantages of simultaneous multi-drug therapy in controlling drug-resistant epilepsy. Further research on the mechanisms of BZDs resistance and optimization of treatment strategies can not only improve the therapeutic effect of drug-resistant epilepsy but also provide a scientific basis for the development of antiepileptic drugs in the future.

## 1 Introduction

Epilepsy is one of the most common neurological disorders and is characterized by recurrent seizures caused by abnormal neuronal discharges in the brain (1). Drug-resistant epilepsy (DRE) refers to the seizure frequency that lasts for 3 months or longer and is not effectively controlled under appropriate drug treatment, or the number of seizures increases significantly in a short period under appropriate treatment (2). It is estimated that more than 70 million people worldwide are affected by epilepsy, and nearly a third of them have DRE (3, 4). As the population ages and lifestyle changes, the prevalence of DRE is likely to rise further (5). The financial burden and care needs of such patients are much higher than those of patients with drug-sensitive epilepsy, imposing heavy pressure on global medical systems and social resources (6).

Benzodiazepines (BZDs) are a class of drugs that exert sedative, anxiolytic, and antiepileptic effects by acting on *γ*-aminobutyric acid (GABA) receptors in the central nervous system (7). These drugs are widely used as a first-line treatment for acute seizures, especially in status epilepticus (SE) (8, 9). Benzodiazepine-resistant epilepsy refers to the condition in which patients develop resistance to BZDs after long-term use, leading to ineffective treatment or a significant decline in therapeutic efficacy.

This suggests that resistance to BZDs, as a first-line drug for treating acute seizures, is a challenge for patients with epilepsy, and it is essential to seek the most effective treatment methods and strategies (8). Recent studies have focused on the elucidation of the mechanisms of drug-resistant epilepsy in phenytoin and carbamazepine (9, 10). This article reviews the definition, mechanisms of resistance, and treatment methods of benzodiazepine-resistant epilepsy. An in-depth study of the mechanism is helpful to the development of clinical treatment, and looks forward to new treatment methods and potential drug development of benzodiazepine-resistant epilepsy, hoping to provide scientific guidance for the research of benzodiazepine-resistant epilepsy.

## 2 Mechanisms of action and resistance of benzodiazepines

### 2.1 Action mechanism of benzodiazepines

BZDs are positive allosteric modulators of GABA_A_ receptors and regulate the allosteric function of the receptor by binding to the BZDs site of the receptor, the interface site between the *α*2/α3 and γ2 subunits of the GABA receptor (11). The conformation of the receptor changes, which increases the affinity of the binding site of GABA (between *α* and *β* subunits) to endogenous GABA and enhances the amplification effect of the GABA signal (12). Even if the regulation of BZDs on the receptor is positively correlated with the concentration of endogenous GABA (11), it can also cause a strong inhibitory response to low concentrations of GABA. Moreover, BZDs increase the probability of chloride (Cl^−^) channel opening, prolong the open duration of Cl^−^ channels, and enhance Cl^−^ influx, resulting in neuronal hyperpolarization and further inhibition of neuronal firing. Activation of GABA receptors causes Cl^−^ influx through Cl^−^ channels, leading to hyperpolarization of the neuronal membrane potential, thereby reducing neuronal excitability (13).

### 2.2 Resistance mechanisms of benzodiazepines

#### 2.2.1 Drug target-related mechanisms

##### 2.2.1.1 Decreased GABA_A_ receptor number

Long-term use of BZDs enhances receptor endocytosis, leading to a decrease in the total amount of GABA_A_ receptors, especially in the key brain areas involved in drug action (e.g., hippocampus and cortex) (14). For example, chronic exposure of neurons to diazepam activates the downstream Ca^2+^ signaling cascade of GABA_A_ receptors, leading to a gradual decrease in cell surface GABA receptors through dynamin-dependent endocytosis. The endocytosis process is regulated by protein kinases and phosphatases that determine the phosphorylation status at specific sites of the β and *γ* subunits of the receptor. When specific residues are dephosphorylated by protein phosphatase 1, 2A, or calcineurin—that is, when the GABA_AA_R γ2 subunit is dephosphorylated at Ser327 in cortical neurons—receptor internalization is promoted, removing it from the postsynaptic membrane. This ultimately triggers disaggregation of inhibitory synapses, resulting in a decrease in the total amount of GABA receptors (15, 16).

##### 2.2.1.2 Decreased GABA_A_ receptor activity

Astrocytes take up glutamate and convert it to glutamine by the enzyme glutamine synthetase (GS), which is catalyzed by glutamate decarboxylase to form GABA in GABAergic neurons (17). Decreased GS activity leads to decreased GABA production. GABA concentration is insufficient in the synaptic cleft, and the modulation of receptors by BZDs is positively correlated with the concentration of endogenous GABA_A_ (11), impairing the activity of GABA receptors.

##### 2.2.1.3 Altered GABA_A_ receptor structure

Changes in GABA_A_ receptor subunit composition and gene mutations can lead to drug-resistant epilepsy.

The mechanisms underlying changes in receptor subunit composition mainly include the decrease of γ2 subunit expression and the substitution of *α* subunit. The γ2 subunit is the binding site of BZDs, and its reduction directly affects the binding and action of BZDs. In SE, GABA_A_ receptors containing synaptic γ2 subunits selectively transport and relocalize to the cell interior, leading to loss of synaptic inhibition and development of BZDs resistance in early SE (18). In drug-resistant refractory epilepsy, the expression of the α1 subunit is decreased, while the α4 or α5 subunits, which are less sensitive to BZDs, are relatively increased (19). The α1 subunit may inhibit dynamin-mediated endocytosis (20), which is enhanced by decreased α1 subunit expression, thereby reducing the number of cell surface GABA_A_ receptors.

GABA-A receptor variants can be classified into loss-of-function (LOF) and gain-of-function (GOF) types, which affect receptor structure and function through distinct mechanisms. LOF variants (e.g., GABRA1, GABRB2, GABRB3, GABRG2) lead to reduced surface expression, assembly defects, or decreased GABA-induced currents, resulting in weakened inhibitory neurotransmission and abnormal synchronous discharges in the cortico-thalamic network. These patients typically respond well to GABA-enhancing agents such as BZDs (21–24). In contrast, GOF variants (e.g., GABRB3, GABRD) increase receptor sensitivity to GABA or induce constitutive activation, often with mild expression defects. These variants primarily cause excessive inhibition in specific neural circuits like the thalamic reticular nucleus, leading to network desynchronization and abnormal rhythms, including spike-and-wave discharges (SWDs). For such patients, GABA-enhancing drugs may exacerbate symptoms or trigger paradoxical reactions. Thus, both LOF and GOF variants can contribute to DRE (25, 26).

#### 2.2.2 Synaptic and neural network remodeling

Cytokines released after brain injury can induce astrocyte dysfunction, leading to impaired ion and neurotransmitter homeostasis, which increases susceptibility to seizures (27, 28). Synaptic and neural network remodeling mainly includes three aspects: the involvement of neuroinflammation, the functional decline of inhibitory synapses, and the enhancement of excitatory synapses.

##### 2.2.2.1 Involvement of neuroinflammation

Seizures can cause neuroinflammatory responses, and neuroinflammation plays an important role in epilepsy (29). “Neurogenic neuroinflammation” refers to the synthesis and release of pro-inflammatory molecules triggered by enhanced neuronal activity in astrocytes and other brain cells (27, 30, 31). Astrocytes and microglia contribute to neuronal hyperexcitability and seizures (27, 32).

Microglia release specific signaling molecules, such as TNFα, IL-1α, and complement component subunit 1q (C1q). These signaling molecules induce the generation of the A1 reactive astrocytes. The proinflammatory phenotype (A1) exhibits IP3 R2-mediated Ca^2+^ hyperactivity, which leads to excessive production of proinflammatory cytokines and reactive oxygen species, thereby exacerbating neural damage (27, 33–35). In SE and chronic epilepsy stages, IL-1β produced by microglia can increase extracellular glutamate concentration by inhibiting astrocytic glutamate transporters. It can also suppress GABA-mediated Cl^−^ influx into neurons, resulting in increased neuronal excitability (29). The release of the inflammatory cytokine TNF-*α* causes endocytosis of GABA_A_ receptors, causing a reduction in surface GABA_A_ receptors and a decrease in inhibitory synaptic strength. TNF-α effectively induces neuronal cell death through apoptosis or necroptosis signaling pathways (33), regulating neuronal circuit homeostasis, which may exacerbate excitotoxic damage caused by neuronal injury and further disrupt synaptic network balance (36).

Astrocytes play an important role in the regulation of synaptic function and plasticity. Astrocytes release gliotransmitters, such as ATP and D-serine, which directly modulate synaptic strength and plasticity (33). In addition, under the control of TGFβR and IL-1β signaling, inflammatory astrocytes express high levels of matrix metallopeptidase family proteins, which can promote extracellular matrix remodeling and further promote pathological synaptic plasticity (27).

##### 2.2.2.2 Functional decline of inhibitory synapses

Glial cells that synthesize GABA, such as astrocytes, release GABA through non-vesicular mechanisms (e.g., channel-mediated release), serving as a source of extracellular GABA in the brain (37, 38). The reduction of intercellular GABA may be due to decreased GABA synthesis. GS participates in the process of GABA synthesis (17, 39). Beta-amyloid, oxidative stress, nitrosative stress, tissue inflammation, and several environmental factors can easily lead to the downregulation or inhibition of GS in astrocytes (17). The decrease in GS activity promotes an increase in extracellular glutamate regulated by astrocytes and reduces GABA production in neurons due to decreased glutamine supply (27).

##### 2.2.2.3 Enhancement of excitatory synapses

In the constant electrical activity of neurons and interneurons in SE, two major neurotransmitters, glutamate and GABA, are released in excess in the mammalian brain. Sustained hyperactivity and overstimulation trigger changes in synaptic receptor function, in which the AMPA and NMDA types of glutamate receptors on major cells are functionally enhanced, leading to further augmentation of excitatory synaptic activity; at the same time, the function of GABA_A_ receptors is inhibited, which weakens the inhibitory effect of BZDs on neuronal hyperexcitability (40). The dominant role of these two receptor functions in the development of epilepsy has not been clearly defined and still requires further investigation (27). Excessive glutamate not only increases the likelihood of seizures but also causes neuronal loss through its excitotoxic properties, further aggravating the pathological remodeling of neural networks (17).

#### 2.2.3 Metabolism-related pathway gene variants

Genetic variations in the CYP450 enzyme system may influence the rate of BZDs metabolism, thereby altering the effective concentration of the drug in the body. The CYP3A subfamily is primarily responsible for the metabolism of anti-seizure medications (ASMs) (41), with the CYP3A4 gene being involved in the 1′-hydroxylation of BZDs. Mutations in this gene may increase the efficiency of this process, resulting in a shortened drug half-life or reduced drug efficacy, potentially affecting the metabolism of ASMs and resulting in lower drug concentrations in the body, which can impact the therapeutic effects of the drug (42, 43).

#### 2.2.4 Drug efflux-related mechanisms

**Figure 1:**
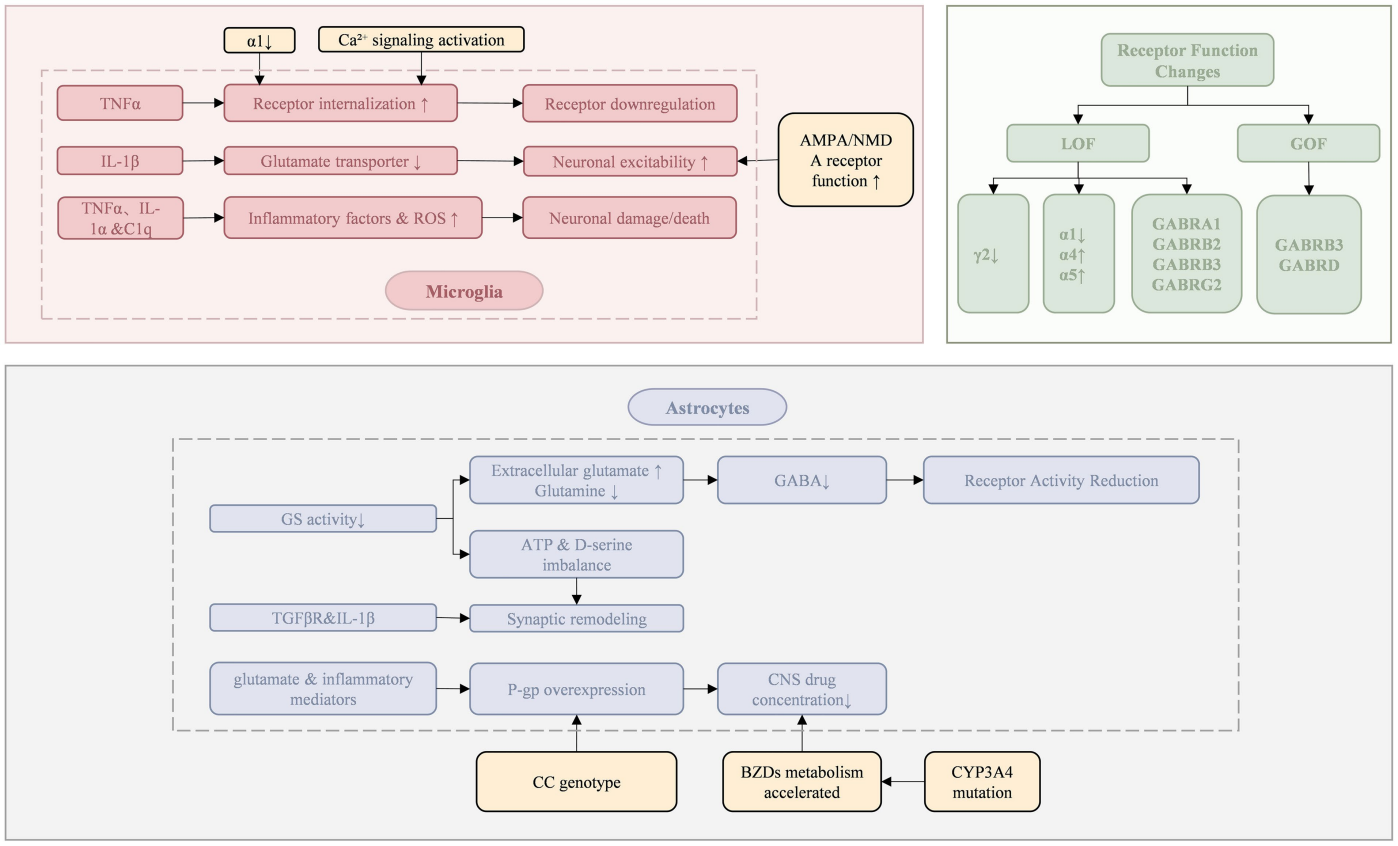
Resistance mechanisms of benzodiazepines.

The transporter hypothesis suggests that overexpression of P-glycoprotein (P-gp) in the blood–brain barrier (BBB) is a key mechanism of resistance in ASMs (44). As an efflux transporter that influences the distribution of drugs in the central nervous system, P-gp actively exports BZDs and other drugs from brain tissue (45). P-gp is encoded by the ABCB1 gene, and the CC genotype of the ABCB1 C3435T polymorphism is associated with elevated P-gp protein levels and increased activity (46). Up-regulation of P-gp expression can significantly reduce the concentration of the drug in the central nervous system, thereby diminishing its efficacy. In patients with drug-resistant epilepsy, glutamate and inflammatory mediators released by perivascular astrocytes may further exacerbate this drug-resistance phenomenon by inducing the up-regulation of P-gp (27). In addition to P-gp, other efflux transporters, such as multidrug resistance-associated proteins (MRPs), may efflux some metabolites of BZDs, such as glucuronic acid (47) (see Figure 1).

## 3 Treatment methods

BZDs, such as diazepam and lorazepam, are widely used in the treatment of epilepsy (48). Because of its rapid onset of action and significant effect, BZDs have become the first-line treatment for acute seizures and SE. However, the problem of drug resistance is common in clinical practice, which not only limits the effectiveness of drugs but also increases the complexity of patients’ conditions and the difficulty of treatment. To solve the treatment problem of BZDs-resistant epilepsy, a variety of innovative approaches have been developed in recent years.

### 3.1 Pharmacological treatment

#### 3.1.1 Medication use optimization

The optimal use of drugs includes two aspects: drug combination and individualized treatment.

Early identification of refractory epilepsy syndrome and drug-resistant epilepsy is crucial for formulating treatment strategies. For patients with a potential risk of drug resistance and easy-to-develop drug-resistant epilepsy, the combination therapy of ASMs can be attempted early. The following key considerations should be taken into account when implementing combination therapy: (1) the selected ASMs should have distinct mechanisms of action; (2) pharmacodynamic studies should demonstrate a synergistic enhancement of efficacy between the drugs; (3) pharmacokinetic evaluations should indicate no significant drug interactions or, at the very least, no adverse synergistic effects; (4) adverse reactions should not be synergistic or cumulative (49, 50). If combination therapy does not yield clinical benefits, it is advisable to transition to monotherapy or adjust the combination regimen to achieve an optimal balance between therapeutic efficacy and tolerability of adverse effects.

Different drugs have different mechanisms of action in the treatment of epilepsy (51). BZDs inhibit seizures by regulating neuronal inhibition, while drugs such as levetiracetam, lamotrigine, and sodium valproate inhibit seizures by regulating neuronal excitability, and drugs such as phenytoin, sodium valproate, and levetiracetam inhibit seizures by regulating voltage-gated ion channels (52). The combination of drugs is usually two drugs with different mechanisms, such as the combination of GABAA receptor agonists and NMDA receptor antagonists. In the synaptic GABA_A_ receptor loss and NMDAR increase caused by status epilepsies (SE) (53), BZDs restore the inhibitory effect by stimulating the remaining GABA receptors, as long as a sufficient number of receptors remain present on the postsynaptic membrane. However, this only partially solves the problem, as the increase in functional NMDA receptors and the resulting excitotoxicity have not been addressed. Thus, treatment of receptor transport abnormalities induced by SE requires at least two drugs: a GABA_A_ receptor agonist, such as BZDs, and an NMDA receptor antagonist (54). Their combination may not only terminate RSE but also reduce or eliminate some of its long-term consequences: neuronal damage, spatial memory deficits, and seizures. At the same time, their therapeutic index is improved by synergistic interaction (53).

The advantage of combination therapy is that it can enhance the efficacy of drugs, reduce adverse reactions, and reduce the occurrence of drug resistance. However, attention must be given to the different mechanisms of action of the drugs to avoid interactions that could increase drug side effects. BZDs such as lorazepam or diazepam are usually used first for rapid onset of treatment. When patients become resistant to BZDs, BZDs can be combined with drugs such as phenytoin sodium, valproate sodium, and levetiracetam, which is an effective strategy for the treatment of BZDs-resistant epilepsy (55).

The standard approach for treating SE is sequential multidrug therapy, where a second drug is administered after the failure of the first drug, and a third drug is given after the failure of the second (53). Typically, BZDs are followed by another anticonvulsant, then another novel anticonvulsant, followed by general anesthesia, and, after a few failed episodes of anesthesia, ketamine or another less commonly used drug (54, 56). The advantage of this approach is that it maximizes the delivery and minimizes drug toxicity for responders. However, the downside is that sequential polytherapy requires time, as each treatment must wait until the failure of the previous one before starting the next. Delay delivery of the second drug by at least 30 min, and delay delivery of the third drug by at least 1 h (53). During this time, untreated receptor alterations induced by the initial drug may worsen, potentially complicating the efficacy of subsequent targeted therapies (e.g., ketamine). If the first-line treatment is BZDs, the increased internalization of GABA receptors may lead to an upregulation of untreated NMDARs, resulting in heightened neuronal excitability. We should consider concurrent multidrug therapy to reverse the effects of receptor trafficking early before it becomes irreversible (54). One study demonstrated that simultaneous multidrug therapy was far more effective than sequential monotherapy or higher-dose midazolam in reducing electroencephalography power (EEG power) and stopping SE (53).

Individualized treatment is an important way to treat drug-resistant epilepsy, which needs to be carried out under the guidance of specialists. In individualized treatment, detailed etiological diagnosis and analysis of drug metabolism characteristics are needed first, and then individualized treatment plans are designed according to the specific conditions of patients, and the most suitable drugs are selected for treatment to achieve the best therapeutic outcome. In cases where BZDs fail to control seizures at their maximum recommended dosage, or when patients exhibit intolerable adverse effects, alternative therapeutic options may include the administration of intravenous anesthetic agents such as thiopental sodium or propofol. Additionally, other mechanisms of ASM should also be considered (57). Furthermore, in individualized treatment, the dose and administration regimen of drugs need to be adjusted according to the specific condition of the patient. For example, for patients with rapid drug metabolism, it is necessary to increase the drug dose or shorten the dosing interval to ensure therapeutic efficacy. Genetic testing can help predict the rate of BZDs metabolism and guide the selection of personalized drug doses. In patients with a low-active CYP3A4 allele, the dose of BZDs may need to be adjusted to avoid toxicity or inefficiency issues (58); Tariquidar is a P-glycoprotein inhibitor that can be used in patients with genotypes associated with P-gp overexpression (59).

#### 3.1.2 Novel drug treatments

##### 3.1.2.1 New drugs

New drugs, such as other Positive allosteric modulators of GABA_A_ receptors, include neuroactive steroids and other drugs. Neuroactive steroids are at various stages of clinical development, such as Ganaxolone, Zuranolone, LYT-300, Sage-324, PRAX 114, and ETX-155. Other medications include nonsteroid compounds (e.g., GRX-917, a TSPO-binding site ligand), *α*2/3-preferring BAER-101, *α*2/3/5-preferring SAN711, and KRM-II-81, raising new therapeutic prospects for this highly leveraged drug target in neurology and psychiatry (60, 61).

Ganaxolone is a neurosteroid analogue that exerts antiepileptic effects by enhancing the inhibitory function of GABA_A_ receptors. Unlike BZDs that bind to the *γ* subunit of GABA_A_ receptors, ganaxolone acts at the neurosteroid binding site, mainly at the *β*-α subunit interface of GABA_A_ receptors, thereby avoiding the common dependence and resistance issues of BZDs. Ganaxolone has been proven to be effective in various refractory epilepsy syndromes, especially those related to CDKL5 deficiency syndrome (62). Its intravenous formulation is also under development and is expected to become a potential treatment option for SE. However, except for BZDs, most drugs that enhance GABA function usually exacerbate the occurrence of absence epilepsy in the brain. Ganaxolone, as a GABA enhancer, also exhibits similar characteristics. It enhances the tonic inhibition mediated by GABA_A_ receptors, particularly in regions such as the thalamus and visual cortex. These regions are the key structures highly related to the thalamocortical circuit of absence epilepsy. Therefore, although ganaxolone shows broad therapeutic potential in many epilepsy types (63), it should be used with caution in patients with absence epilepsy, as its mechanism of action may instead induce or exacerbate seizures (64, 65).

Cenobamate (CNB) is a novel antiepileptic drug characterized by a dual mechanism of action: inhibition of persistent sodium currents and positive modulation of GABA_A_ receptor function. In recent years, its potential efficacy in drug-resistant genetic generalized epilepsy (GGE) has attracted increasing attention. Preclinical animal studies have demonstrated that CNB exhibits significant anticonvulsant effects across multiple models of generalized seizures, including the GAERS rat model, the 6 Hz psychomotor seizure model, and seizure models induced by maximal electroshock (MES) and pentylenetetrazol (PTZ). CNB was shown to reduce SWDs and the duration of absence seizures, suggesting broad-spectrum efficacy against various types of generalized seizures (66, 67).

##### 3.1.2.2 New drug delivery forms

Novel drug delivery modalities include changing from tablets to nasal sprays, oral mucosal delivery, and intravenous formulations. Inhaled BZDs are a group of central nervous system drugs delivered by aerosol or vapor. They are mainly used for the rapid treatment of anxiety attacks, panic attacks, and SE (68). Compared with traditional oral or intravenous forms, inhaled BZDs have the advantages of faster onset of action and patient compliance. For example, midazolam aerosol has been approved in some countries for the emergency management of both out-of-hospital and in-hospital seizures (69). As a derivative of Levetiracetam, Brivaracetam (BRV), which enhances inhibitory neurotransmission by positively regulating GABA_A_ receptors, has a higher affinity and faster onset and can be used in the form of intravenous injection. It provides a new option, especially when rapid control of seizures is needed (70).

##### 3.1.2.3 Improved drugs

Modified drugs, such as Fosphenytoin sodium, are suitable for SE. It is a water-soluble prodrug of phenytoin that is rapidly converted to Phenytoin in the body (71). Fosphenytoin sodium is widely used in the treatment of acute seizures, especially in SE, because of its excellent water solubility, safety, few side effects, and the ability to quickly reach therapeutic concentrations by intravenous or intramuscular injection (72).

### 3.2 Adjuvant therapy

Several other treatments can aid in the treatment of epilepsy. Neuromodulation techniques such as vagus nerve stimulation (VNS) send electrical signals to the vagus nerve through implantable devices to modulate neuronal activity in the brain, thereby reducing seizures (73). Additionally, non-invasive VNS is an emerging method (74). Acupuncture is a traditional Chinese medicine therapy that regulates the body’s functions by stimulating specific acupoints to alleviate epilepsy symptoms and reduce the side effects of drugs (75). For example, auricular acupoint therapy with transcutaneous auricular VNS is used to treat epilepsy (76).

Simultaneously, some traditional Chinese medicine can be used as an auxiliary treatment for epilepsy, such as Curcumae Longae Rhizoma, Scorpio, Acori Tatarinowii Rhizoma, Uncariae Ramulus *Cum* Uncis, and Ganoderma, etc., with sedative and anticonvulsant effects (77). The ketogenic diet (KD) has shown remarkable anti-epileptic and disease-modifying characteristics by restricting glucose utilization, promoting ketogenesis, and regulating astrocyte metabolism. Its mechanisms include inhibition of glycolysis, such as the use of 2-deoxy-D-glucose, regulation of glutamate signaling, and reduction of abnormal expression of astrocyte-related genes. Furthermore, the ketogenic diet also blocks the epileptogenic process in epigenetic pathways by increasing the level of adenosine and down-regulating the expression of adenosine kinase (ADK), it also reduces the progression of epilepsy and related risks, such as sudden unexpected death in epilepsy (SUDEP) and drug resistance (27, 78).

In rare cases, surgical treatment can be considered (79). If the acute phase of epilepsy is triggered by an obvious brain trauma or structural lesion, such as a brain tumor or vascular malformation, surgery can be used to deal with the underlying cause, thereby helping to control the seizures (80, 81). If a patient presents with refractory SE, does not respond to multiple medications, and is life-threatening, surgery may be considered as a last resort, and such surgery usually involves callosotomy or local excision of the lesion (82). However, these methods are not a substitute for conventional epilepsy treatment.

### 3.3 Frontier treatment

Stem cell therapy, gene therapy, anti-inflammatory therapy, and small molecule drug research provide new insights for the cutting-edge treatment of epilepsy.

#### 3.3.1 Stem cell therapy

Stem cell-based therapies offer a new avenue for long-term control of DRE through regenerative properties. Its mechanisms include specific cell substitution, rescue and repair of degeneration cells, reorganization of synapses, modulation of the secretion of neurotransmitters, and beneficial neurotrophic factors. Stem cell transplantation can not only alleviate seizures, but also inhibit the progression of epilepsy, prevent the development of chronic epilepsy, and improve cognitive function, showing many therapeutic potentials. It has been used to treat epilepsy in preclinical animal studies and clinical trials (83).

#### 3.3.2 Gene therapy

Optogenetics and chemogenetics have shown great potential in the research and treatment of DRE. Gene therapy selectively manipulates excitatory or inhibitory neurons to regulate abnormal neural activity at the molecular level. Optogenetics uses light to control the excitability of specific neuronal populations and can be used in a closed-loop paradigm to activate light sources only when a seizure is detected. Chemical genetics relies on the modification of endogenous receptors or modified chimeric receptors in response to exogenous ligands (79). These two techniques have successfully suppressed seizures in various epilepsy models in mice, rats, and monkeys, showing significant promise in the study of neuropathological states and the treatment of epilepsy (84). Additionally, significant breakthroughs have been made in the study of neuropeptide Y (NPY) in gene therapy. By overexpressing NPY in the brain using viral vectors, seizures can be significantly suppressed through the activation of Y2 and Y5 receptors. Once the obstacles of gene therapy are overcome, the endogenous neuroregulatory system can be used to treat epilepsy (85).

#### 3.3.3 Anti-inflammatory therapy

Anti-inflammatory therapy reduces seizure frequency, provides neuroprotection, and improves cognitive function by targeting inflammatory molecules induced by reactive astrocytes. Some anti-inflammatory drugs, such as Anakinra, have shown efficacy in refractory status epilepticus and pediatric epileptic encephalopathy, particularly in patients for whom traditional drug treatments are ineffective (27). The various inflammatory pathways in neuroinflammation include neurodegeneration, neurogenesis, glial proliferation, axonal injury and sprouting, dendritic plasticity, blood–brain barrier (BBB) damage, neuroinflammatory processes, extracellular matrix reorganization, and neuronal molecular structure reorganization. Only a few molecular mechanisms have been targeted to prevent or modify the epilepsy development or phenotype, and no such therapy has as yet been developed, which can provide new ideas for antiepileptic drugs (86).

#### 3.3.4 Small molecule drugs

Small molecule drugs are still the core field of epilepsy treatment. As a Negative Allosteric Modulator (NAM), methyl-6,7-dimethoxy-4-ethyl-*β*-carboline-3-carboxylate (DMCM) can reduce GABA_A_ receptor activity at low concentrations. However, at high concentrations, especially when coexisting with flumazenil, DMCM can turn into a positive modulator and enhance GABA-induced current. This dual-action mechanism offers new strategies for exploring drug combination approaches, potentially optimizing the therapeutic effects of epilepsy treatment in specific situations (87).

Although technical and clinical translation challenges remain, these therapies are expected to become important treatments for DRE in the future.

## 4 Summary

Benzodiazepines are one of the main drugs for the treatment of epilepsy, but the problem of drug resistance seriously restricts their clinical application (8). The development of benzodiazepine resistance is the result of the joint efforts of many factors, and its mechanism covers many aspects, such as the drug target-related mechanisms, synaptic and neural network remodeling, metabolism-related pathway gene variants, and the mechanism related to drug efflux. Current treatment options for DRE include optimization of drug use, new drug therapy, and adjuvant therapy. Although some progress has been made in the mechanism analysis and treatment strategies in recent years, there are still many unresolved issues to be explored. Future studies should focus on combining multidisciplinary techniques to reveal the underlying mechanisms of BZDs resistance and develop more precise and personalized treatment methods to improve the clinical outcomes of DRE patients. Furthermore, with the rapid development of neuropharmacology and molecular biology, new drugs, stem cell therapy, and gene therapy intervention strategies may provide breakthroughs to solve the problem of BZDs resistance, thus promoting the treatment of neurological diseases into a new era (79, 83).
